# Special Issue: Conductive Polymers: Materials and Applications

**DOI:** 10.3390/ma13102344

**Published:** 2020-05-20

**Authors:** César Quijada

**Affiliations:** Departamento de Ingeniería Textil y Papelera, Universitat Politècnica de València. Pza Ferrándiz y Carbonell, E-03801 Alcoy (Alicante), Spain; cquijada@txp.upv.es; Tel.: +34-966-528-419

**Keywords:** polyacetylene, polyaniline, polypyrrole, PEDOT-PSS, copolymers, charge transport models, silica gel composite, carbon composite, antimicrobial, drug release, sensors, adsorption

## Abstract

Intrinsically conductive polymers (CPs) combine the inherent mechanical properties of organic polymers with charge transport, opto-electronic and redox properties that can be easily tuned up to those typical of semiconductors and metals. The control of the morphology at the nanoscale and the design of CP-based composite materials have expanded their multifunctional character even further. These virtues have been exploited to advantage in opto-electronic devices, energy-conversion and storage systems, sensors and actuators, and more recently in applications related to biomedical and separation science or adsorbents for pollutant removal. The special issue “Conductive Polymers: Materials and Applications” was compiled by gathering contributions that cover the latest advances in the field, with special emphasis upon emerging applications.

Intrinsically conductive polymers (CPs) are a fascinating family of organic materials that can be easily synthesized with a large diversity of chemical structures and a wide variety of micro- and nano-morphologies in order to obtain tailored macroscopic physical and chemical properties [1]. The breakthrough discovery of polyacetylene, the first conducting polymer, by Heeger, MacDiarmid and Shirakawa in 1977 (jointly awarded the Nobel Prize in Chemistry for 2000), opened up a completely new field of research, tracking the boundaries between chemistry and solid-state physics. Since then, interest in this intriguing class of polymers has expanded incessantly to become a well-established area of highly dynamic, multidisciplinary research.

From a chemical viewpoint, CPs are π-conjugated organic polymers, that is to say, compounds with their skeletal carbon atoms linked by both σ-bonds and the extended overlap of π-electron orbitals. As a result, neutral CPs show a semiconductor electronic structure with a completely filled π-band (valence band) and an empty π*-band (conduction band), separated by an energy gap of the order of 1 eV. The intrinsic conductivity arises from *doping*, i.e., when electrons are withdrawn or injected onto the conjugated polymeric chain, while the overall electroneutrality is retained by the incorporation of counter ions, the so-called *dopants*. The doped state is achieved by simple oxidation (p-doping) or reduction (n-doping) reactions, leading to the formation of delocalized charged structural defects (polarons, bipolarons, solitons) that are energetically located within the energy gap and work as charge carriers. Thus, the electrical conductivity of these polymers can be tuned over the full range from insulation to metallic by facile and reversible chemical or electrochemical doping/de-doping.

Jointly with simple control of the doping level, the electronic structure of CPs can be “engineered” by designing novel chemical structures at the molecular level through judicious synthetic chemical or electrochemical strategies, thus enabling a set of desired and tunable optical, electronic and redox/electrochemical properties. This concept has evolved to produce a vast repertory of chemically diverse, lightweight and flexible tailor-made polymer structures, showing attractive properties for a broad spectrum of applications with high technological impact, many of them reaching the marketplace. Some examples are their use as key materials in thin-film transistors, light-emitting diodes and solar cells, electrochromic displays, (bio)sensors and actuators, secondary batteries and supercapacitors or artificial muscles, just to cite a few [1]. Over the past few decades, some other new applications in biomedical science and tissue engineering, enhanced solid-phase extraction or as adsorbent/ion-exchange materials for environmental issues have emerged as promising growth areas.

In spite of the obvious advantages of CPs over their inorganic semiconducting and metallic counterparts in terms of chemical diversity, tunable conductivity, low density and cost and flexibility, much work is still to be done in order to overcome their inherent limitations regarding solubility/processability, conductivity and long-term stability. For this, the development of CP-based hybrid composites with enhanced properties and controlled shape and morphology has become a flourishing area of discovery within the field. Typical examples are CP nanocomposites with a variety of carbon nanomaterials, metal oxide nanoparticles, or hydrogel inorganic matrices.

The Special Issue “Conductive Polymers: Materials and Applications” was launched to cover the latest advances and developments in the synthesis, characterization, structure–properties relationship and applications of intrinsically conductive polymers, with particular attention given to novel functionalized polymer/copolymer structures or novel CP–inorganic composite materials and their use in emergent applications. The nine articles included in the issue touch different aspects of the goals pursued. A brief summary of their main achievements and conclusions is given below.

In undoped trans-polyacetylene, bond alternation has been widely recognized as the obvious consequence of the existence of two-equivalent degenerate ground states arising from the Peierls instability. Hudson [2] critically reviewed the available experimental studies (X-ray diffraction (XRD), nuclear magnetic resonance (NMR), Raman or infrared (IR) data) that support the existence of bond alternation and concluded that their results are compromised by the presence of finite chains or finite conjugation segments or other ambiguities. The author proposed a novel synthetic route with the aid of urea inclusion complexes to produce fully extended all s-trans polyacetylene, free of finite-chain polyene impurities.

In recent years, the application of conducting polymer-based materials to biomedical science and healthcare has been the subject of intense research activity. The papers by Robertson et al. [3] and Shah et al. [4] fall within this category. In the former, the authors investigated the activity of polyaniline (PANI) and poly(3-aminobenzoic acid) (P3ABA) as potential antimicrobial agents against *Escherichia coli* and *Staphylococcus aureus*. Both CPs showed high bactericidal action in suspension, but their efficacy was depressed when applied to agar (an adsorbent surface) and especially to styrene-ethylene-butyrene-styrene films (a non-adsorbent surface). This behavior was attributed to the decreasing contact occurring between bacterial cells and CPs on going from solution to adsorbent surfaces to non-adsorbent films. The remaining activity of P3ABA-containing surfaces was reported to be superior to that published for triclosan, a popular antimicrobial agent, which encouraged the use of this polymer in cost-effective antimicrobial surfaces to break pathogen transmission pathways in hospitals. On the other hand, Shah et al. [4] reported the use of polypyrrole (PPY)-based coatings loaded with clinically relevant drugs as systems for switchable drug delivery. The anti-inflammatory dexamethasone phosphate (DMP) and the antibiotic meropenem (MER) were loaded as the anionic dopants. Analysis by energy-dispersive X-ray (EDX) spectroscopy, Fourier transform infrared (FTIR) spectroscopy, ultraviolet–visible (UV–vis) spectroscopy, XRD and electrochemical techniques (cyclic voltammetry (CV) and electrochemical impedance spectroscopy (EIS)) confirmed the presence of the drugs in the films. The authors showed that the electrochemically-triggered release of drugs in vitro was enhanced relative to the passive delivery from non-stimulated samples. The higher improvement observed for MER was attributed to its higher hydrophilicity and polar character, which makes it more responsive to electrically stimuli within the PPY matrix.

The application of CPs and their composites to the detection of biologically active molecules of interest in medical diagnosis, food or plant analysis is the subject of another three articles. A feature paper by Dkhili et al. [5] reported the electrosynthesis of novel, chemically stable aniline-piperazine copolymer films that exhibited reversible hydroxyl-ketopiperazine redox transitions between the well-known redox processes of polyaniline. The new copolymer material showed reproducible linear response and high sensitivity for the analysis of dopamine and ascorbic acid, thus being potentially applicable to amperometric sensors. Hybrid materials consisting of silica gel matrices modified with CPs have found interesting applications in analytical science. Djelad et al. [6] described the synthesis of SWCNT-modified silica films by electro-assisted deposition from sol-gel precursors, and their further modification by reactive electrochemical insertion of PEDOT-PSS from the monomer and dopant constituents. The resulting SWCNT@SiO_2_–PEDOT-PSS composite doubled the electroactive surface area and exhibited a threefold increase in the heterogeneous rate constant of ferrocene, a common electron shuttle in electrochemical biosensors. Sowa et al. [7] modified commercial silica gel particles with PANI by chemical polymerization. Confocal Raman microscopy revealed that PANI preferentially deposited inside the inner pores of the silica particles. The authors optimized an experimental procedure for the extraction and quantification of triterpenic acids from plant samples by using PANI-modified silica gel as the sorbent in dispersive and matrix solid-phase extraction (d-SPE and MSPD) coupled to diode array detection high-performance liquid chromatography (DAD-HPLC).

Finally, the special issue also illustrates the use of CP-based composites as adsorbent materials for the removal of environmentally hazardous chemicals. In a series of two papers, Muhammad et al. [8,9] examined the adsorption behavior of PANI-Fe_3_O_4_ composites for the removal of cationic Basic Blue 3 (BB3) [8] and anionic Acid Blue 40 (AB40) [9] dyes from aqueous solution. The composite materials were fully characterized by scanning electron microscopy (SEM), FTIR, EDX, UV and XRD [8]. The adsorption of BB3 and AB40 obeyed Langmuir and Freundlich isotherm models, respectively, while kinetics was of the pseudo-second order. PANI-Fe_3_O_4_ composites showed enhanced adsorption capability for BB3 when compared to the individual components and other typical low-cost adsorbents. This effect was associated with an increase in surface area and pore volume of the hybrid material. Instead, the composite material was a less efficient adsorbent for AB40 than pure PANI, probably because repulsive forces from Fe_3_O_4_ oxygen lone pairs offset the electrostatic attraction between oppositely charged sites of PANI and the dye. Quijada et al. [10] electro-synthesized PANI-activated carbon cloth (ACC) composites in a filter-press cell. Based on SEM, X-ray photoelectron spectroscopy (XPS), N_2_ adsorption, thermal analysis, CV and direct current (DC) conductivity data, the authors suggested that thin polymer films containing a small amount of phenazine/phenoxazine segments formed within the micro- and mesopores of the carbon fibers, thus showing enhanced conductivity and pseudocapacitance. In heavily loaded PANI-ACC, a nanofibrous thick coating developed which caused strong pore blocking, diminished surface area, and loss of conductivity. Composites with moderate PANI loadings showed promoted pseudo-second order adsorption rate of Acid Red 27 from aqueous solution, which was related to the electrostatic interaction between dye-negative sites and positive N sites in acid-doped PANI.
